# Mechanisms of Body Weight Fluctuations in Parkinson’s Disease

**DOI:** 10.3389/fneur.2014.00084

**Published:** 2014-06-02

**Authors:** Andrea Kistner, Eugénie Lhommée, Paul Krack

**Affiliations:** ^1^Movement Disorder Unit, Department of Psychiatry and Neurology, University Hospital Grenoble, Grenoble, France; ^2^Unité 836, Équipe 11, INSERM, Grenoble Institut des Neurosciences, Grenoble, France; ^3^Joseph Fourier University, Grenoble, France

**Keywords:** Parkinson’s disease, body weight, eating behavior, DBS, dopamine, binge-eating disorder

## Abstract

Typical body weight changes are known to occur in Parkinson’s disease (PD). Weight loss has been reported in early stages as well as in advanced disease and malnutrition may worsen the clinical state of the patient. On the other hand, an increasing number of patients show weight gain under dopamine replacement therapy or after surgery. These weight changes are multifactorial and involve changes in energy expenditure, perturbation of homeostatic control, and eating behavior modulated by dopaminergic treatment. Comprehension of the different mechanisms contributing to body weight is a prerequisite for the management of body weight and nutritional state of an individual PD patient. This review summarizes the present knowledge and highlights the necessity of evaluation of body weight and related factors, as eating behavior, energy intake, and expenditure in PD.

## Introduction

Parkinson’s disease (PD) is a chronic neurodegenerative disease with diffuse α-synuclein deposits in the neural system (1). The most prevalent symptoms in early disease are mainly due to progressive degeneration of the dopaminergic nigrostriatal and mesocorticolimbic pathways with motor (akinesia, rigidity, tremor) and non-motor (apathy, anxiety, depression) symptoms (2). The disease is also characterized by the presence of non-motor vegetative symptoms explained by a synucleinopathy of central and peripheral vegetative system (3) and in its advanced stages by dementia, which correlates with cortical deposits of alpha-synuclein (intracellular Lewy bodies and Lewy neurites) (4). On top of the dopaminergic system, serotonergic, noradrenergic, and cholinergic nuclei in the brainstem projecting to the cortex are also impaired by the diffuse synucleinopathy which starts in the lower brainstem. Typically, the first alpha-synuclein deposits are found in the vagal nerve with a gastroparesis and constipation starting before the first motor symptoms and leading the patients to consult before a diagnosis of PD can be made (5). According to the clinical stage, body weight of a given patient may considerably change during the course of the disease raising the risk for both disease-related under-nutrition and treatment-related overweight.

Body weight is determined by many factors including genetic, epigenetic, metabolic, and environmental components and under physiological conditions homeostatic behavioral adaptations protect against weight gain as well as weight loss (6).

However, regulation of body weight seems to be more effective in response to weight loss than to weight gain (7). Weight gain is the result of a positive energy balance, which means that energy intake exceeds expenditure, resulting in accumulation of fat. Although this equation seems rather simple maintaining a constant body weight is a complex physiological process comprising internal and external, homeostatic and hedonic, and neurological and metabolic factors. The fine regulation of these systems is hindered by the «*obesogenic*» environment characterized by increased availability of large amounts of palatable and energy-dense foods and presence of powerful food cues, together with minimal physical activity. The result is the increasing prevalence of obesity in western communities (8).

The situation of PD patients should be seen against this background with additional factors in relation to the severity of the disease and dopaminergic treatment: these factors include (1) perturbation of hypothalamic metabolic regulation, (2) alteration of energy expenditure (EE) (*through tremor, rigidity, dyskinesia, physical activity including hyperactivity, sleep disorders*), and (3) alteration of intake (i.e., *perturbation of swallowing, gastrointestinal disorders, alteration of eating behavior*).

The aim of this review is:

- To highlight pathophysiological mechanisms implicated in nutrition and leading to body weight fluctuations in PD.
- To summarize available data about body weight fluctuations in PD (literature until January 2014).
- To link observed body weight fluctuations to possible mechanisms in order to improve future patient care of PD patients.

## Physiological Mechanisms

### Homeostatic control of food intake

**Figure 1:**
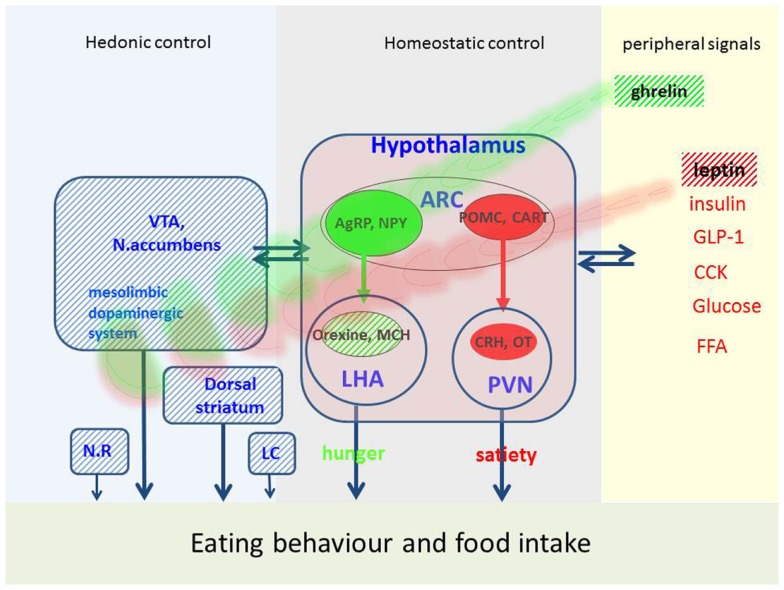
**Possible factors implicated in eating behavior and food intake in PD**. Eating behavior is regulated by hedonic, homeostatic, and peripheral signals. This figure represents the main mechanisms, which are disturbed in PD (hatched areas) and may affect eating behavior. VTA, ventral-tegmental area; ARC, arcuate nucleus; NR, nucleus raphe; LC, locus coeruleus; PVN, paraventricular nucleus; LHA, lateral hypothalamus; AgRP, agouti-gene related peptide; NPY, neuropeptide Y; MCH, melanin-concentration hormone; POMC, proopiomelanocortin; CART, cocaine-and-amphetamine-regulated transcript; CRH, corticotropin-releasing hormone; OT, oxytocin; GLP-1, glucagon-like peptide; CCK, cholecystokinin; FFA, free fatty acids.

Homeostatic control of food intake and energy metabolism is assured by a network of several hypothalamic nuclei (Figure 1) [for review see Ref. (9–11)].

#### Central pathway of hunger

The neuronal «pathway of hunger» includes neurons in the arcuate nucleus containing orexigenic peptides (i.e., agouti-gene-related peptide, neuropeptide Y). These neurons project to the lateral hypothalamus called «hunger center» which contains neuronal populations producing orexigenic peptides like melanin-concentration hormone and orexins (also called hypocretin). The name orexin refers to the Greek term for appetite (orexis). Orexin increases appetite, arousal, and spontaneous physical activity and therefore plays an important role in energy balance. Loss of orexin leads to loss of appetite and a reduced metabolism. Orexins are thought to provide a link between energy balance, behavioral arousal, and reward (10, 12) and play a role in thermogenesis (13).

Both orexin and melanin-concentration hormone neurons are selectively lost in PD which is correlated with the clinical stage and severity of disease (14–16). Thus, deficiency of orexigenic peptides could contribute to the weight changes in PD. However, the exact mechanisms in PD are complex and far from being understood.

#### Central pathway of satiety

Another neuronal population in the arcuate nucleus is encoding satiety. These neurons produce numerous anorexigenic peptides, such as α-melanocyte-stimulating hormone, derived from a common precursor, called pro-opiomelanocortin (POMC). Together with their receptor, these peptides constitute the melanocortin system which plays an important role in energy homeostasis. Interactions of this system with dopamine neurons in the nucleus accumbens are described (11). Another central satiety signal is cocaine-and-amphetamine-regulated transcript (CART) which was also shown to interact with hedonic circuits and dopamine (11). These peptides project to the «satiety center» located in the paraventricular nucleus which contains other anorexigenic peptides such as corticotropin-releasing hormone (CRH) and stimulation of these neurons reduces appetite. Today, a large number of central signals are identified and the list is still growing [for review see Ref. (9–11)].

#### Peripheral signals

This network assures a bidirectional homeostasis in response to peripheral signals reflecting the actual nutritional stage. The «satiety pathway» is activated by several factors, i.e., gastric dilatation, intestinal peptides liberated in postprandial state (cholecystokinin, glucagon-like peptide, peptide YY), metabolites as fatty acids and glucose and hormones as insulin and leptin. The latter is a hormone synthesized and liberated by adipose tissue. Serum levels of leptin reflect the degree of adipose tissue and high levels reduce food intake. Accordingly, in PD patients who lost body weight leptin tends to be low (16–18) and increases when body weight increases, e.g., after DBS surgery (19). In mice lacking D2 receptors, an enhanced hypothalamic leptin signaling has been shown (20) arguing for an alteration of this mechanism in PD which could explain weight loss in spite of low leptin levels.

Hunger and appetite may be induced by ghrelin, which is the only peripheral hunger signal thus far identified. Ghrelin is a peptide synthesized and liberated from the gastric mucosa in fasting state (10). In addition to actions in the hypothalamus, ghrelin stimulates appetite via receptors located in mesolimbic circuits (9). In PD, plasma ghrelin levels are low and paradoxically correlated with BMI (17). A reduced postprandial ghrelin response was shown in early stages of PD (21) which is not modified by dopamine treatment or acute STN-stimulation (22, 23). Thus, low levels of ghrelin may contribute to weight loss in PD (24). Six months after STN-stimulation, ghrelin was shown to increase (25) which is coherent with the concomitant weight gain (see later).

### Dopaminergic control of eating behavior

The hypothalamic control of food intake is modulated by the dopaminergic system and both systems are modulated by homeostatic orexigenic and anorexigenic signals such as ghrelin and leptin (26, 27). Dopamine and the dopamine D2 receptor play a central role in motivated behavior including feeding behavior (28, 29). However, the role of the dopaminergic system in feeding behavior is very complex and not completely understood. It seems to exert different actions in separate circuits and in the pattern of release (phasic versus chronic release) (26, 30).

Exposure to food and food-related cues results in an activation of the mesolimbic dopamine system and especially the projection from the ventral tegmental area to the nucleus accumbens [for review see Ref. (10, 30)]. This led to the hypothesis that the mesolimbic dopamine system mediated pleasure associated with eating [for review see Ref. (26, 27, 30)]. This idea is strongly challenged since it was shown that dopaminergic depletion of nucleus accumbens does not blunt the hedonic response to pleasant food and dopamine is not required for “liking” of food (28, 31). In line with these results, dopamine-deficient mice still demonstrate a marked preference for sucrose over water (31).

Instead, dopamine is necessary for motivational processes, referred to as “incentive salience” or “wanting” [for review see Ref. (28)]. Accordingly, increasing the synaptic dopamine by inhibition of the DA transporter or administration of amphetamine increase the motivation for high effort activities [for review see Ref. (30)]. This motivation can be measured in laboratory animals with running activity or lever pressing in order to acquire foodstuff. Lack of mesolimbic dopamine reduces the activity of rodents to acquire foodstuff, for example running activity or working to get access to food (26, 28, 32). On the other hand, when palatable food is abundant and may be acquired without effort, food intake of dopamine-deficient rodents remains stable or even increases (32).

Other dopaminergic pathways are implicated in eating-related behaviors: in dopamine-deficient mice, restoration of dopamine in dorsal striatum rescues feeding in these animals that otherwise would die of starvation (26). It seems that a regulated dopamine release in dorsal striatum is essential for normal feeding in mice (33) and humans (34). The dopamine release in the dorsal striatum following feeding correlated with the experienced pleasure in healthy humans (35). Obese people have low striatal dopamine D2 receptor availability (36), and low dorsal striatal presynaptic dopamine synthesis capacity was correlated with overeating behavior in a PET study with healthy volunteers (37).

These observations led to the “reward deficiency hypothesis for overeating.” According to this hypothesis, overeating may be considered as a “therapy” of low dopaminergic state leading to weight gain and obesity [for review see Ref. (29)]. However, as stated by Berridge et al. (28), the decrease of D2 receptors in obesity could also be a downregulation following overeating.

On the other hand, too much dopamine signaling was shown to inhibit feeding in mice, demonstrated with non-specific dopamine receptor agonists, DAT inhibitors, or amphetamines which increase the synaptic dopamine via an inhibition of presynaptic dopamine reuptake (26). In healthy adults, the amphetamine and DAT inhibitor methylphenidate reduces eating and food intake by one-third (38, 39) and weight loss was shown in PD patients treated with methylphenidate (40). In hypothalamic pathways, dopamine inhibits feeding (28), and a tonic inhibition of orexin-producing neurons in the lateral hypothalamus by dopaminergic hotspots in the nucleus accumbens has been described [for review see Ref. (29)]. However, in PD treatment with dopamine agonists may induce in some cases compulsive eating behavior leading to weight gain (41). This eating behavior is often referred to as “binge-eating disorder (BED)” and is considered to be an impulse control disorder (ICD). The underlying mechanism is thought to be an activation of mesolimbic dopaminergic pathways, especially in ventral striatum and nucleus accumbens, mediated by D3 receptors (42).

Taken together the present knowledge, we may conclude:

(1) Dopamine is necessary for motivational salience and efforts linked to alimentation, like shopping and preparing meals.
(2) In physiological conditions, dopamine over-signaling inhibits feeding.
(3) In some cases, a dopamine overstimulation may increase motivation for food leading to a drive to eat, foraging behavior, and overeating (craving or binge-eating), known to occur in PD patients under treatment with dopamine agonists (see later).
(4) In hypo-dopaminergic state, taste perception and appreciation of the foodstuff (liking) is maintained. Thus, as feeding does not require any effort and highly palatable food is easily available, snacking can be maintained even in apathetic patients who lost motivation for any other physical activity.
(5) Thus, both hypo- and hyperdopaminergic traits may lead to overeating with subsequent weight gain. Eating behavior in both cases may be different.

### Serotonergic and noradrenergic modulation of energy metabolism and appetite

Other aminergic systems such as serotonergic or noradrenergic systems are mutually connected with the hypothalamus and may influence homeostatic metabolic regulation. Both systems are affected by alpha-synucleinopathy (43). The noradrenergic locus coeruleus (LC) as well as serotonergic neurons express high amounts of orexin receptor and dense orexin fiber projections (44). Loss of LC neurons had been described in PD (45). In the 6-OHDA rat model, degeneration of LC produces a transient drop in body weight which could be reversed by DBS-STN (46). This had led to the hypothesis that weight variations in PD could be modulated by noradrenergic interaction between LC, STN, and hypothalamus (47).

Serotonin plays a role in eating behavior and high cerebral levels may improve mood, depression, and sleep (48). Cerebral serotonin biosynthesis is favored by its precursor, the essential amino acid tryptophan in the presence of dietary carbohydrates. This mechanism is triggered via the insulin response which enhances cerebral uptake of tryptophan (49). Tryptophan is a constituent of many protein-containing foods. The positive effect of carbohydrates, especially those with high glycemic index such as sucrose, on mood could be the reason why efforts to lose weight are doomed to failure in obesity (49) which is often associated with depression (50). Neurodegeneration of the serotonergic system with low levels of serotonin in PD (48) may explain the pronounced preference for all kind of sweets and increased intake of chocolate in PD patients (51).

### Enteric nervous system and gastrointestinal disorders

Gastrointestinal functions are regulated by the enteric nervous system, a neuronal network organized in two plexuses, myenteric and submucosal, which control gut motility and secretion (5).

In PD, the enteral nervous system is affected by alpha-synucleinopathy which may explain the high incidence of gastrointestinal disorders, beginning in pre-motor stages of the disease. The most frequent symptom is chronic constipation affecting up to 89% of PD patients [for review see Ref. (52)]. The primary cause for constipation is impaired peristalsis with slow colonic transit due to neurodegeneration of myenteric neurons, which may be modulated by dopamine (52). In some patients, constipation is secondary to abnormal coordination of the rectoanal reflex with paradoxical contraction of the puborectalis muscle, which leads to defecatory dysfunction (5, 52). Loss of serotonergic neurons in raphe nucleus is thought to be involved in this clinical feature (52). Gastroparesis is characterized by slowed emptying of food into the small bowel leading to postprandial fullness, early satiety, nausea, vomiting, and bloating (53). Gastrointestinal disorders affect the quality of life and may limit food intake thus contributing to mal- and under-nutrition in PD (24).

## Parkinson’s Disease

### Weight changes in pre-motor PD

In prospective American cohort or case–control and Chinese epidemiological studies, a decrease of body weight was reported several years prior to diagnosis (5 pounds in the 4 year preceding diagnosis) (54–57) (Table 1). On the other hand, large Finnish and Japanese cohort studies reported a *weight gain* in pre-diagnostic PD (58, 59). The same result was found in the Honolulu Heart Program which included Americans of Japanese origin (60). No association between PD and BMI before or at disease onset was reported for the Greece EPIC population (61), the UK-based general Practice Research Database (62), and in Italian case–control studies (63, 64). As degeneration of the dopaminergic system begins years before diagnosis (65), BMI variation may reflect a dysregulation of dopaminergic control of eating behavior rather than modification of energy metabolism in pre-motor stages of the disease. Apathy, depression, and anxiety are frequent in *de novo* PD (66, Bichon et al., personal communication) and eating disorders may also appear in response to these negative emotional state. In the general population, a strong association between depression and overweight has been described (50, 67) which may be due to sub-threshold eating disorders described as “emotional eating” (68), increased “snacking” (69), or increased sweet preference (70). Alterations of eating behavior have been described in *de novo* PD, prior to treatment (Bichon et al., personal communication).

**Table 1:** **Body weight modification in pre-motor PD**.

Reference	Study type	Population	*n*	Origin	PD cases	Result
Chen et al. (54)	Prospective cohort	NHS^a^, HPFS^a^	160,000	USA	468	Weight loss
Logroscino et al. (56)	Prospective cohort	Harvard Alumni Health Study	10,812	USA	106	Weight loss
Cheshire and Wszolek (55)	Case–control study		100 + 100	USA	100	Weight loss
Ma et al. (57)	Prospective cohort	Rural population Lixian	16,488	China	464 (85 analyzed)	Weight loss
Hu et al. (58)	Prospective cohort	Cross-sectional population surveys	47,156	Finland	526	Weight gain
Ikeda et al. (59)	Prospective cohort	Check up in health care center	20,000	Japan	24	Weight gain
Abbott et al. (60)	Prospective cohort	Honolulu Heart Program	7990	USA/Japanese origin	137	Weight gain
Kyrozis et al. (61)	Prospective cohort	EPIC^a^ Study	26,173	Greece	120	No association
Becker et al. (62)	Retrospective database analysis	Database	5,000,000	UK		No association
Ragonese et al. (63)	Case–control study		318 + 318	Italy	318	No association
Scigliano et al. (64)	Case–control study	Clinical records of newly diagnosed PD	178 + 533	Italy	178	No association

*^a^NHS, National Health Study; HPFS, Health Professional Follow-up Study; EPIC, European Prospective Investigation into Cancer and Nutrition*.

Furthermore, disturbance of smell and taste may alter preference for foodstuff. In fact, nutritional factors have been correlated with “PD-risk”: in a Chinese study, meat consumption was inversely associated with PD (57) and in a Japanese study, dietary glycemic index was inversely correlated with PD-risk (71). An association of dietary milk protein with PD-risk is established for large prospective US cohorts (72), but a case–control study in Japan could not confirm this association (73). As dietary patterns are very different between Asian and western populations, these findings might reflect cultural variations of eating behavior in pre-motor PD. A recent meta-analysis found significant negative associations with PD for smoking, coffee drinking, and alcohol consumption which may represent a modification of preferences in early PD (74).

### Prevalence of malnutrition in PD

Weight loss in PD has been reported since the first publication of James Parkinson in 1817. A recent meta-analysis on BMI in PD reported a lower BMI of PD patients than controls (pooled difference: −1.73 kg/m^2^), which is related to disease severity (75). Average weight loss is about 3.6 kg 8 years after diagnosis (54) or 6 kg in one decade (76). Both fat mass and lean body mass were reported to be reduced in PD patients who lost weight (18, 77). It should be outlined that a lower average BMI does not mean that many PD patients are at risk for malnutrition. In spite of a decline of body weight, during disease progression patients may be overweight (see later). Prevalence of underweight depends on the used assessment tool and ranges from 0 to 24% (4–5% in the control group), while 3–60% of PD patients were reported to be at risk of malnutrition (77). However, the use of the mini nutritional assessment, a valid nutrition assessment tool, resulted in malnutrition rates of only 0–2% while 20–34% were at risk of malnutrition (77). Malnutrition is associated with disease severity (78).

### Predictors of weight loss in PD

#### Increased energy expenditure

**Figure 2:**
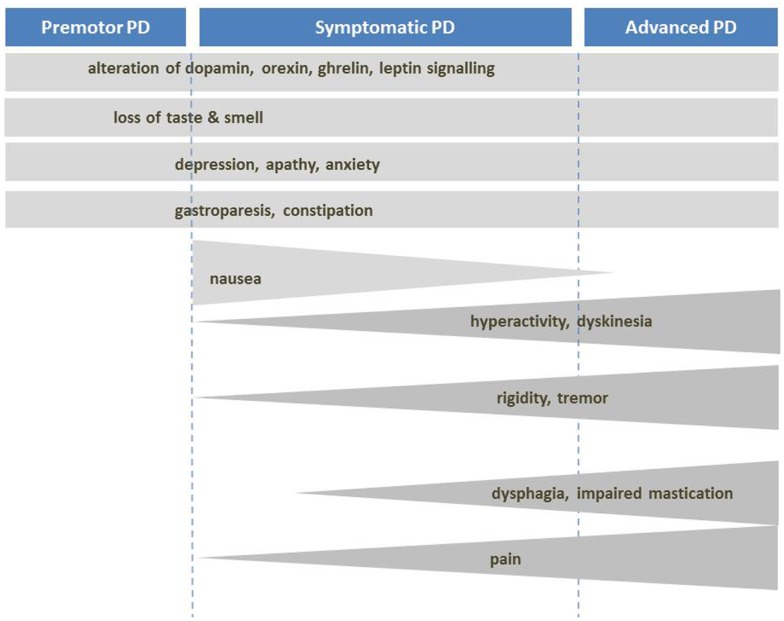
**Mechanisms of body weight loss in PD**. Mechanisms of body weight loss in Parkinson’s disease according to the stage of the disease. Factors with important contribution to weight loss are dark grey.

Despite eventual weight loss, PD patients increase their energy intake by about 350 kcal/day, mainly due to increased carbohydrate intake (54, 79) (Figure 2). This suggests that weight loss is induced by increased energy expenditure (EE). Indeed, metabolic studies had shown that resting EE is increased in PD (about 20–51% of control subjects) in ON and OFF-medication state. The main factors for this increase seem to be dyskinesia and rigidity (80–87). Consequently, when patients with severe dyskinesia were excluded, resting EE was not increased (87–89). Dyskinesia and rigidity as well as tremor may be considered as spontaneous physical activity, like standing or fidgeting. In healthy volunteers, it was shown that spontaneous physical activity may account for EE up to 700 kcal/day (90). If this is not compensated by energy intake, weight loss is inevitable.

#### Impaired homeostatic regulation of energy metabolism

Taken into account that weight loss may take place before the onset of motor symptoms, other disease-related factors should be considered. As described above, several disorders of the hypothalamic regulation are known in PD, namely a massive loss of hypothalamic orexin-producing neurons, occurring prior to the onset of drug treatment (15, 16, 91). As orexin is involved both in appetite and spontaneous physical activity (92), its decrease may contribute to a decrease in food intake and physical activity. Weight loss could be promoted by impaired bioenergetics due to mitochondrial dysfunction, as shown in a mouse model of PD. In this model, which has a loss-of-function mutation of the mitochondrial protein kinase (PINK1) causing a genetic form of PD, significant weight reduction occurred in pre-motor stage (93).

Ghrelin, the gastric «hunger hormone» is reduced in PD and has even been considered as a potential biomarker of the disease (21). This could be due to impaired gastric mobility and contribute to weight loss in all stages of the disease [for review see Ref. (24)]. Furthermore, evidence from studies with rodents indicate that hypothalamic leptin signaling (which acts as a satiety signal) might be enhanced in PD (20).

#### Impact of dopaminergic treatment

As described above, dopamine has anorectic effects in the hypothalamic arcuate nucleus leading to a suppression of appetite and food intake (28). Likewise, substances which increase the synaptic dopamine by inhibition of the presynaptic dopamine transporter like amphetamine have anorectic effects (38).

In addition, dopaminergic treatment, especially apomorphine, may induce nausea and vomiting thus limiting energy intake, mainly on introduction of treatment (94). However, in the long-term, the treatment is mostly tolerated and may induce dyskinesia and behavioral side effects including physical hyperactivity both leading to increased EE.

#### Other factors

Other factors include impairment of gastrointestinal function (dysphagia, delayed gastric emptying, constipation, mal-absorption), disturbed hand–mouth-coordination, and other motor symptoms limiting activities of daily living or a decline in cognitive functions (95–99). Medical conditions such as infection, bone fractures, and malignant diseases may be other factors (99). Levodopa is an amino acid and its intestinal absorption and cerebral uptake competes with dietary amino acids thus may be impaired by dietary protein. A low-protein diet may increase its bioavailability. Patients with severe postprandial OFF-periods are often advised for a “protein-redistribution diet” which is based on the protein restriction throughout the day whereas the daily protein ration is consumed at dinner. These diets may further worsen the nutritional state of the patient (100). Some authors reported a link of PD with impaired glucose homeostasis (101, 102) but presently there is no clear evidence in this context [for review see Ref. (103)] and a recent meta-analysis confirmed a lack of relationship between PD and diabetes (104).

### Weight gain and overweight in PD

**Figure 3:**
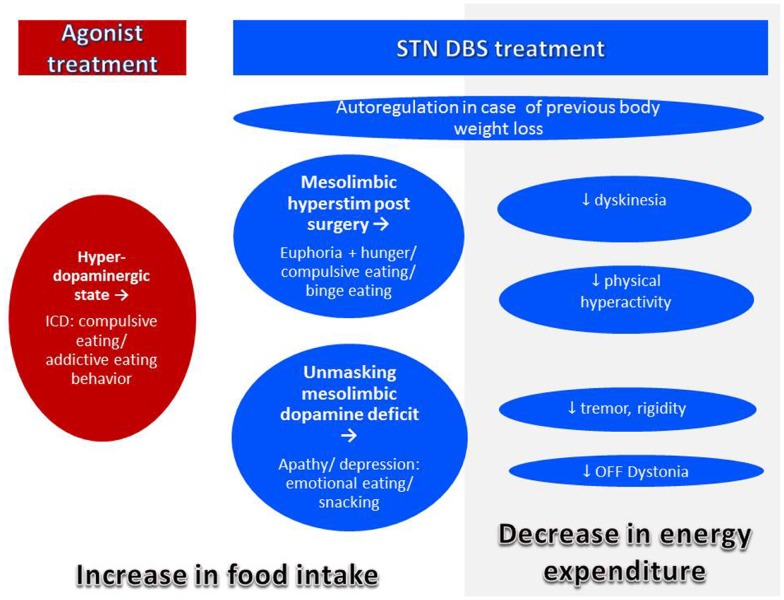
**Mechanisms of body weight gain in PD**. Body weight gain may occur with treatment by agonist or DBS. Both agonist and DBS treatment may lead to modifications of eating behavior leading to increased food intake. In addition, with successful DBS treatment, energy expenditure decreases by improvement of symptoms leading to body weight gain even in the absence of eating disorders.

In the pre-levodopa era, PD was a disease of malnutrition and even 20 years ago, it was rare to encounter obese PD patients (98) (Figure 3). Today, in spite of a decreased average BMI, individual PD patients may be overweight (75). In fact, prevalence of overweight in PD was reported to be about 60% in Italy (105) and 50% in Germany (80). In France and USA, more than 50% of the patients selected for DBS, i.e., patients with advanced disease suffering from motor complications presented an overweight with a BMI >25 kg/m^2^ (106–108). These data are close to the prevalence of overweight and obesity in the general population in western countries which is between 50 and 70% (109). We can state that modern pharmacotherapy together with overall increase in overweight in modern society seems to have changed the phenotype of the PD patients. Modern treatment for PD as agonists and DBS may have an additional impact.

#### Weight gain with dopamine replacement therapy

As stated above, levodopa may be considered as an anorexigenic agent. In contrast, dopamine replacement therapy (DRT) and in particular D2/D3-selective dopamine agonists may lead to behavioral changes, ICDs, and among them changes in eating behavior, referred to as BED (110) and explained by an activation of the “reward system” mediated by D3 receptors (42). Eating disorders were described in up to 15% of patients treated with dopamine agonist leading to weight gain and sometimes to obesity (110–120). Nocturnal eating is frequent and related to sleep disorders in patients treated with agonists (121). Other alterations of eating behavior are described as “insatiable craving,” “compulsive overeating,” “binge-eating” with uncontrollable and compulsive traits as common feature (41, 122–126). In patients with mood fluctuations, eating of snacks occurred more likely in “ON” state while they experienced euphoria (127). Lowering or discontinuation of dopamine agonist may reverse the symptoms (41).

#### Weight gain after surgery for PD

Weight gain after pallidotomy for PD was first published in 1953 (128–130) (Table 2). Since then many authors reported increased body weight after surgery for PD, mostly STN-DBS (131, 132), but also stimulation of GPi (107, 133–135). In case of STN-DBS, weight gain is present in the majority of patients, is rapid, that is occurs mainly within the first 3 months (131, 136, 137). In the long-term, body weight tends to stabilize in many patients (138, 139) and some patients may lose weight again, but mean weight remains higher than before surgery (136, 140). In individual patients, excessive weight gain leading to obesity was described (106). Weight gain seems to be independent of target (GPi or STN) and procedure (lesion, stimulation, uni-or bilateral) (107, 134, 141). However, in a recent study, STN was more associated with weight gain as GPi (142). In any case, weight gain is achieved by a positive energy balance for which several mechanisms may play a role.

**Table 2:** **Weight gain in PD after surgical treatment: possible mechanisms and predictive factors**.

Reference	*N* enrolled	FU (m)	*n* Patients with WG^a^	Mean increase of BMI (kg/m^2^)/body weight (kg)	Target and surgical procedure	Findings and predictive factors
Guiot and Brion (128)	47	12	20	5–16 kg	Pallidotomy	WG in the first months after surgery
Lang et al. (129)	40	12	14	13.6 kg	Pallidotomy	Greater ease of feeding, altered eating behaviour, reduced dyskinesia
Moro et al. (132)	7	9	7	8 kg	STN-DBS	Increased appetite
Ondo et al. (130)	60	12	49	4 kg	Pallidotomy unilateral	WG predicted by improvement of motor score, most pronounced during first months
Gironell et al. (133)	27	12	26	4.7 kg	STN-DBS, GPi-DBS, pallidotomy	WG predicted by improvement of dyskinesia, motor score, pre-op weight
Krack et al. (131)	42	60	39	4 kg	STN-DBS	WG occurs in the first months after surgery and remains stable after 1 year
Barichella et al. (136)	30	12	29	3.1 kg/m^2^, 9.3 kg	STN-DBS	Improvement of dyskinesia score
Macia et al. (150)	19	13 ± 8	18	4.7 kg/m^2^/9.7 kg	STN-DBS	Decreased REE with unchanged DEI, increase of fat mass
Tuite et al. (144)	27	6	22	1.2 kg/m^2^/4.5 kg	STN-DBS	No significant weight gain in the immediate post-operative period, weight gain occurred after stimulator was activated
Novakova et al. (138)	23	45	23	3.3 kg/m^2^/9.4 kg	STN-DBS	Body weight tended to stabilize in long-term
Montaurier et al. (86)	24	3	Data not given	1.1 kg/m^2^/3.4 kg (men), 1.0 kg/m^2^/2.6 kg (women)	STN-DBS	Low improvement of UPDRS motor score, EE decreased but did not correlate with weight gain
Bannier et al. (106)	22	16	20	2.2 kg/m^2^/5.5 kg	STN-DBS	Low pre-operative body weight, low improvement of UPDRS motor score
Guimarães et al. (176)	57	3	41	3 kg/m^2^	STN-DBS	Nutritional intervention prevents weight gain
Walker et al. (108)	39	12	33	0.4 kg/m^2^/4.3 kg	STN-DBS unilateral	No correlation with UPDRS or LED, no association with the side of unilateral DBS
Sauleau et al. (135)	46	6	37	5.7 kg (STN), 1.7 (GPi)	STN-DBS vs. GPi-DBS	WG in STN-DBS > GPi-DBS, association of WG with dyskinesia in GPi-DBS, no change of food intake
Moghaddasi and Boshtam (137)	15	3	Data not given	6 kg	STN-DBS	Rapid weight gain after DBS
Strowd et al. (107)	99	24	Data not given	2.3 kg	STN-DBS, VIM-DBS	WG greater in VIM-DBS
Escamilla-Sevilla et al. (19)	14	6	12	1.2 kg/m^2^/5.5 kg	STN-DBS	Increase of leptin without expected decrease of NPY, correlation with higher stimulation voltages
Locke et al. (134)	44	6	31	2.3 kg	STN-DBS unilat. vs. GPi-DBS unilat.	No difference in WG between STN and GPi, no correlation with clinical parameters
Lee et al. (141)	43	24	Data not given	5 kg (male), 4 kg (female)	STN-DBS uni + staged STN-DBS bilateral	No statistical difference in WG
Serranová et al. (160)	20	34	18	8 kg	STN-DBS	WG correlates with arousal ratings of appetitive stimuli
Novakova et al. (172)	27	12	24	5.2 kg	STN-DBS	Decrease of cortisol levels, no other changes
Foubert-Samier et al. (149)	47	12	37	2.7 kg/m^2^/7.2 kg	STN-DBS	High pre-operative motor score
Markaki et al. (25)	23	6	17	6 kg	STN-DBS	WG associated with changes of ghrelin, leptin, and NPY. Decrease of cortisol
Ružicka et al. (152)	20	18	19	6.9 kg	STN-DBS	WG correlated with electrode position distance to three ventricles
Jorgensen et al. (145)	7	12	Data not given	4.7 kg	STN-DBS	Decreased DEE with unchanged DEI, weight gain = fat mass
Mills et al. (142)	31 + 30	>12	Data not given	+0.53 kg/m^2^ STN, −0.14 kg/m^2^ GPi	STN-DBS, GPi-DBS	WG target-specific (STN > GPi)

*^a^WG, weight gain; IAPS, international affective picture system; REE, resting energy expenditure; DEI, daily energy intake; NPY, neuropeptide Y; DEE, daily energy expenditure; LED, levodopa-equivalent dose; STN-DBS, subthalamic nucleus deep brain stimulation, if not otherwise stated bilateral*.

##### Normalization of body weight after previous weight loss

A compensation of previous weight loss in underweight patients is normal and desirable. In humans as in animals, a period of starvation results in hyperphagia related to the extent to which body fat was previously depleted. Therefore, a drive to overeat seems to be part of a regulatory system to restore fat mass (6). The phenomenon of “rebound adiposity” in PD after STN-DBS was described by Dulloo and Montani (143). Accordingly, weight gain after DBS in PD may be correlated with the pre-operative weight, and initially underweight patients take more weight (106, 133). However, weight gain often exceeds previous weight loss by far (144). Thus, additional mechanisms have to be considered.

##### Reduction of energy expenditure with unchanged intake

After STN-DBS, a significant decrease (7–13%) of daily EE was reported (86, 145). A decrease of EE of 13% without adaptation of intake would lead to a weight gain of 20 kg after 1 year (145). This decrease of EE after successful STN-DBS may be explained by:

- a reduced resting EE (87) following improvement of rigidity and tremor,
- a reduction of levodopa-induced dyskinesia (146),
- reduction of OFF-period dystonia (147),
- a reduction of levodopa-induced behavioral hyperactivity (121),
- an improvement of sleep pattern and nocturnal hyperactivity (121, 148).

Accordingly, correlations of weight gain with reduction of motor symptoms, reduction and severity of OFF-periods, LEDD reduction, and reduction of levodopa-induced dyskinesia (133, 136, 149, 150) had been described. Despite improved motor response and decreased EE after DBS, 80% of the patients do not increase their physical activity nor change their eating habits (136). This imbalance between intake and expenditure leads inevitably to weight gain, mostly due to an increase in fat mass (86, 106, 150).

##### Direct impact of STN-stimulation on adjacent brain regions

A direct effect of STN-stimulation on the hypothalamus by current diffusion has been hypothesized as indeed the lateral hypothalamus is very close to the medial limbic tip of the STN (151). In a recent study, weight gain after chronic DBS of STN was inversely related to the distance of the contacts from the wall of the third ventricle (152). However, this observation could not distinguish between current diffusion to the hypothalamus or to the mesolimbic part of the STN. In the rat, a lesion of the STN without lesioning the hypothalamus leads to impulsive feeding behavior (153), strongly arguing for a lesioning effect of the mesolimbic STN. The most impressive clinical behavioral changes may be observed immediately after stimulation has been switched on, while patients are still in the hospital. Patients in the immediate post-operative state may experience a euphoric state induced by STN-DBS, characterized by disinhibition, hyperactivity, and increased appetite. This condition spontaneously recovers within few weeks or months and is thought to be linked to both the lesional effect of surgery with an edema of the STN and to the long-term response of mesolimbic effects of dopaminergic medication (154). The most important gain weight occurs in the first weeks and months until stabilizing. The inverse is the case for stimulation settings, which start very low, and then gradually are increased over time in order to avoid behavioral changes (154). The volume of current diffusion in neural tissue does not increase in a linear way with increase of current setting. On the contrary, there is sharp exponential decrease of efficiency with the distance to the electrode (155). If weight gain were to be explained by current diffusion to the hypothalamus, the weight gain should increase over time with increasing stimulation parameters. Furthermore, high-frequency stimulation or lesion of the lateral hypothalamus has anorectic effects, as shown in rats (156) and obese humans, respectively (157). Altogether, these are strong argument against current diffusion to the hypothalamus as the explanation of weight increase after surgery for STN-DBS.

Weight gain in PD patients treated with STN-DBS is accompanied by increasing levels of leptin reflecting the increasing degree of adipose tissue (19, 25). In normal conditions, high leptin levels have an anorectic function on the hypothalamus by downregulating the expression of orexigenic neuropeptide Y in the arcuate nucleus, a mechanism which ensures body weight stability (158). It was shown that in PD patients treated with STN-DBS, neuropeptide Y levels increase despite high leptin levels and it was argued that DBS interferes with the inhibitory action of leptin in the hypothalamus (19, 25). However, 3 or 6 months after STN surgery, basal levels of hormones of the hypothalamic–adrenal, –gonadal, and –somatotropic axis were normal and hypothalamic function in STN-DBS was considered to be normal (159). These observations argue for a direct effect of STN-stimulation to the mesolimbic STN which has an influence on motivation for food intake in rats (153). Accordingly in PD patients with STN-DBS, an increased sensitivity to food reward cues was reported which correlated with post-operative weight gain (160). However, as is the case for other behavioral effects of STN-DBS, some tolerance effect developing over the first months after surgery is likely (154). It is possible that STN-DBS interferes with homeostatic hypothalamic regulation, but not related to current diffusion toward the hypothalamus.

##### Eating disorders

As described above, treatment with D2/D3-specific dopamine agonists may lead to compulsive eating behavior, which disappears after discontinuation of treatment (Table 3). Alteration of eating behavior has also been described in PD patients treated with STN-DBS (113, 121, 161). Due to missing classification and nomenclature, hyperphagia is often classified as BED, the only eating behavior disorder (beside bulimia and anemia nervosa) which is described in DSM-IV. BED includes recurrent and frequent bulimic episodes with lack of control and marked distress. There is no compensatory behavior as vomiting. BED is considered as an ICD (110) and as such is part of scales assessing ICD such as the Questionnaire for Impulsive–Compulsive Disorders in PD (162). However, these scales do not inquire about the frequency and amount eaten and therefore do not allow a BED diagnosis. Consequently, they report high false-positive rates (120). In general, assessment of eating behavior in PD in the literature is not systematic and may range from simple telephone interviews, patients self-reports, retrospective database research of key words to different psychological scales.

**Table 3:** **Eating disorders in PD**.

Reference	Study	*N* total	*N* cases	Prevalence %	Approach	Eating disorder	Disorder related to
Nirenberg and Waters (41)	Case report	–	7	–	Definition of CE and BED, not validated	Compulsive eating BED	Dopamine agonist
McKeon et al. (125)	Case report	–	2	–	Patient self-report	Compulsive eating/night-eating	Dopamine agonist
Giladi et al. (116)	Case–control	193	7	3.6	Structured interview	New onset excessive drive to eat	Dopamine agonist
Miwa and Kondo (127)	Prospective	60	5	8.3	Structured interview with patient/caregiver	Alteration of preferences	Dopamine agonist
Fan et al. (114)	Retrospective	312	1	0.32	DSM-IV and self-reported and telephone interview	BED	Dopamine agonist
Weintraub et al. (110)	Cross-sectional study	3090	132	4.3	DSM-IV, structured interview	BED	Dopamine agonist
Lee et al. (164)	Cross-sectional study	1167	40	3.4	MIDI modified^a^	Compulsive eating	l-DOPA
Khan and Rana (124)	Case report	–	1	–	Patient self-report	Craving and night-eating	Dopamine agonist
Kenangil et al. (117)	Retrospective	554	9	1.6	Semi structured interview	Compulsive eating	Dopamine agonist
Solla et al. (119)	Prospective	349	10	2.9	Definition according to Nirenberg and Waters	BED	Dopamine agonist
Hassan et al. (122)	Retrospective	321	12	3.7	Research of keywords in database	BED	Dopamine agonist
Ávila et al. (111)	Prospective	216	2	1	Questionnaire	Compulsive eating	Dopamine agonist
Vitale et al. (126)	Retrospective	–	12	–	Definition according to Nirenberg and Waters	Compulsive eating	l-DOPA and dopamine agonist
Hinnell et al. (123)	Case report	–	1	–	Patient self-report	Compulsive eating	Dopamine agonist
Lim et al. (118)	Retrospective	200	27	13.5	QUIP (patient or caregiver)	“Eating”	l-DOPA and dopamine agonist
Zahodne et al. (161)	Prospective	96	9	9.3	EDE-Q^a^, EEDDS^a^	BED and sub-threshold BED	Dopamine agonist, STN-DBS
Farnikova et al. (115)	Case–control	46	4	8.7	DSM-IV criteria	BED	Levodopa
Eusebio et al. (113)	Prospective	110	17	15.5	DSM-IV criteria	BED	Dopamine agonist, STN-DBS
Callesen et al. (112)	Retrospective	490	42	8.6	QUIP	“Eating”	Dopamine agonist
Tanaka et al. (120)	Retrospective	93	10, 3	10.8, 3.2	QUIP interview	“Eating,” compulsive eating	Dopamine agonist/levodopa

*^a^MIDI, Minnesota Impulsive Disorders Interview; EDE-Q, Eating Disorder Examination Questionnaire; EDDS, Eating Disorder Diagnostic Scale; QUIP, Questionnaire for Impulsive–Compulsive Disorders in Parkinson’s Disease*.

When employing scales which assess DSM-IV criteria for BED such as the Eating Disorder Examination Questionnaire (EDE-Q), BED is reported to be rather rare in PD: prevalence was about 1% in a small sample of patients (161), comparable with its occurrence in the general population of 1.4% (163). In contrast, prevalence of sub-threshold pathologic eating behavior (“compulsive eating”) was reported to be between 3.4 and 4.5% in PD (110, 164) and seems to increase after STN-DBS (161). We argue that alterations of eating behavior disorders in PD are mostly not BED but comprise a large spectrum of sub threshold pathologic variants of normal eating behavior, described as «snacking», «night-eating», «sweet preferences», «craving», «compulsive eating» which may not all be impulsive. This may explain why in STN-DBS-treated patients, a marked decrease of ICD was described whereas the prevalence of eating disorders decreases less (121) or even increases significantly (113). In fact, DBS-STN was an independent predictor of sub-threshold eating disorders in a small sample of patients (161).

Impulse control disorders are psychiatric conditions characterized by motivation-based behaviors that involve repetitive reward-based activities and loss of control (165). In PD, ICDs are linked to dopamine dysregulation (165) and hyperdopaminergic conditions (121). Successful surgery allows for a marked decrease of dopaminergic treatment (166), leading to disappearance of hyperdopaminergic behavior (gambling, hypersexuality, buying) with exception of eating disorders (113, 121). This condition is often associated with apathy and hypoactivity (121). In these patients, eating may be the only pleasure-generating activity which does not require any effort and is therefore compatible with apathy, which is defined as a decrease in motivation (167) in opposition with ICD which reflects excess motivation oriented toward pleasurable activities (165). In the absence of dopamine, the hedonic response (“liking”) and the perception of taste is conserved (28). Moreover, feeding of palatable food increases dopamine levels in dorsal (35) and ventral striatum including nucleus accumbens (168). Hyperphagia leading to obesity in hypo-dopaminergic conditions such as ADHD had been interpreted as unconscious “self-therapy” in order to normalize mesolimbic dopamine concentrations (169). Thus, hyperphagia could be related to a relative hypo-dopaminergic state which is the case for many PD patients in the post-operative period on chronic DBS when successful stimulation allows for marked decrease in dopaminergic treatment (121, 166). This hypothesis is compatible with a laboratory study which could show that rats previously treated for 5 days with l-DOPA gain 15% more weight than control rats during 12 weeks *ad libitum* feeding. The authors argue that overeating after dopamine withdrawal might be a side effect of dopaminergic stimulation, (170) and this side effect can easily be explained by a downregulation of the dopaminergic system during dopaminergic treatment followed by a withdrawal syndrome. Of note, a withdrawal state in addiction to cocaine, a direct dopamine increasing drug via inhibition of the dopamine transporter, is characterized by progressive apathy over a period of several weeks and its severity correlates with a progressive striatal dopamine depletion (171).

Given the frequency of apathy after STN-DBS, we propose that the vast majority of eating disorders that appear following DBS in PD should therefore not be considered as ICD but interpreted as sub-threshold pathologic behavior in order to compensate for low dopaminergic signaling. In order to differentiate this particular eating behavior from the compulsive eating observed in patients treated with high dose D2/D3 dopamine agonists, we propose to call this behavior “hypo-dopaminergic snacking.”

Hypo-dopaminergic states more rarely occur after GPi-DBS which does not allow for reduction of levodopa. This may explain why weight gain after GPi-DBS on average is less important than after STN-DBS, and mostly due to the reduction of dyskinesia directly related to GPi-DBS (135).

##### Other factors

###### Improvement of gastric function by STN-DBS and the role of ghrelin

STN-DBS can improve the gastric function in PD and thus improve upper gastrointestinal symptoms such as heavy feeling in the stomach, bloating, nausea or feeling sick, and belching (22). This finding may explain the increased levels of the gastric orexigenic peptide ghrelin in PD patients treated by STN-DBS (25) leading to increased hunger. However, the role of ghrelin in STN-DBS remains unproven since three other authors could not confirm increased levels (22, 23, 172).

###### Alteration of the serotonergic system

STN-DBS reduces the firing rate of serotonergic neurons in raphe nucleus (173). As serotonin is involved in control of appetite, this may have an impact on eating behavior and increased snacking of sweet foods may be due to a lack of serotonin.

###### Altered thermogenesis

Centrally-regulated thermogenesis is an important factor in maintaining stable body weight and obesity resistance. Adaptive thermogenesis takes place in brown adipose tissue and the neuropeptide orexin is a key driver (13, 92, 174). Low levels of orexin are common in PD (see above). Although this peptide is investigated in PD mainly with regard to sleep–wake rhythm, orexin deficiency may have an impact on EE and obesity resistance. In fact, PD patients are intolerant to high temperature and drenching sweats is a non-motor symptom which disappears after surgery (175).

### Strategies for maintaining a stable body weight in PD

As weight gain may be desirable or deleterious, the patient’s individual situation should be thoroughly evaluated. Before intervention the following factors should be assessed:

- Actual BMI and previous weight loss, normal weight, previous fluctuations of body weight, and eating disorders.
- Estimation of pre- and post-surgery EE: motor symptoms, dyskinesia, physical activity.
- Actual alimentation, eating habits, and eating disorders.
- Psychological assessment: apathy, depression, hyperactivity.
- Quality of sleep (night-eating disorder).

In DBS patients, nutritional intervention has been shown to be effective (176) and should be performed routinely (95). As weight gain occurs essentially in the first months after surgery, information and dietetic guidance of the patient should start before surgery. As energy requirement is often diminished after successful surgery, an energy-reduced diet should take place and be maintained lifelong. Patients should be encouraged to control their body weight regularly, to supervise their alimentation, and to practice regular physical exercise. These measures should be considered as an adaptation of lifestyle rather than short-time diet.

Recent changes of eating behavior should be taken seriously. Severe hyperphagia with compulsive (craving, binge-eating) or night-eating may improve by discontinuation of agonist treatment. On the other hand, disorders including emotional eating may occur in depressive or hypo-dopaminergic patients treated with STN-DBS. In these patients, a deficit of motivation renders dietary approaches difficult and intervention should first be focused on pharmacological treatment of apathy. Indeed, in selected obese subjects with apathy, it has been shown that treatment with methylphenidate in combination with a weight-loss program was more effective than the weight loss program alone (177). In healthy and obese adults, methylphenidate reduces dietary energy intake by about 20% (38, 39). Thus, alterations of dopaminergic signaling may be an important factor in obesity management of PD.

## Concluding Remarks

In general, body weight gain results from dysregulation of the balance between energy requirement and energy input, the latter is reflected in eating behavior. In PD, dysregulation may be due to alterations of (i) hypothalamic regulation, (ii) energy expenditure, or (iii) dopaminergic signaling. Consequently, different pathomechanisms may account for alteration of eating behavior in PD. Disruption of homeostatic control results in increased appetite and hunger and may be accompanied by compulsive eating behavior. Weight gain despite unaltered eating may argue for reduced energy expenditure. Hyperdopaminergic eating behavior is merely characterized by compulsive and nocturnal eating whereas hyperphagia in hypo-dopaminergic state is part of the hypo-dopaminergic behavior accompanied by apathy and characterized by random snacking and emotional eating.

Understanding the eating behavior may therefore be a window on the underlying factors for weight gain. Any intervention, if pharmacological, behavioral, or nutritional should focus on analysis of the patient’s energy expenditure and a detailed analysis of eating behavior.

## Conflict of Interest Statement

Paul Krack received research grant from Lundbeck, Orkyn, Novartis, Medtronic, LVL, St. Jude; travel costs/honoraria from Euthérapie, Lundbeck, Boehringer Ingelheim, Novartis, UCB, Medtronic, Orkyn, Abbott, Orion, TEVA, Boston Scientific. The other authors declare no conflicts of interest.
